# Cognitive Functions, Neurotransmitter Alterations, and Hippocampal Microstructural Changes in Mice Caused by Feeding on Western Diet

**DOI:** 10.3390/cells12182331

**Published:** 2023-09-21

**Authors:** Raly James Perez Custodio, Zaynab Hobloss, Maiju Myllys, Reham Hassan, Daniela González, Jörg Reinders, Julia Bornhorst, Ann-Kathrin Weishaupt, Abdel-latif Seddek, Tahany Abbas, Adrian Friebel, Stefan Hoehme, Stephan Getzmann, Jan G. Hengstler, Christoph van Thriel, Ahmed Ghallab

**Affiliations:** 1 Leibniz Research Centre for Working Environment and Human Factors at TU Dortmund (IfADo), Ardeystrasse 67, 44139 Dortmund, Germany; custodio@ifado.de (R.J.P.C.); hobloss@ifado.de (Z.H.); myllys@ifado.de (M.M.); hassan@ifado.de (R.H.); gonzalez@ifado.de (D.G.); reinders@ifado.de (J.R.); getzmann@ifado.de (S.G.); 2 Department of Forensic Medicine and Toxicology, Faculty of Veterinary Medicine, South Valley University, Qena 83523, Egypt; abdellatief-shakir@vet.svu.edu.eg; 3 Food Chemistry, Faculty of Mathematics and Natural Sciences, University of Wuppertal, Gaußstraße 20, 42119 Wuppertal, Germany; bornhorst@uni-wuppertal.de (J.B.); weishaupt@uni-wuppertal.de (A.-K.W.); 4 Histology Department, Faculty of Medicine, South Valley University, Qena 83523, Egypt; tahany_abbass@yahoo.com; 5 Institute of Computer Science & Saxonian Incubator for Clinical Research (SIKT), University of Leipzig, Haertelstraße 16-18, 04107 Leipzig, Germany; friebel@izbi.uni-leipzig.de (A.F.); hoehme@uni-leipzig.de (S.H.)

**Keywords:** MASH, neuroinflammation, liver-brain axis, astrogliosis, microgliosis

## Abstract

Metabolic Dysfunction Associated Steatotic Liver Disease (MASLD) is the most common chronic liver disease in Western countries. It is becoming increasingly evident that peripheral organ-centered inflammatory diseases, including liver diseases, are linked with brain dysfunctions. Therefore, this study aims to unravel the effect of MASLD on brain histology, cognitive functions, and neurotransmitters. For this purpose, mice fed for 48 weeks on standard (SD) or Western diet (WD) were evaluated by behavioral tests, followed by sacrifice and analysis of the liver-brain axis including histopathology, immunohistochemistry, and biochemical analyses. Histological analysis of the liver showed features of Metabolic Dysfunction-Associated Steatohepatitis (MASH) in the WD-fed mice including lipid droplet accumulation, inflammation, and fibrosis. This was accompanied by an elevation of transaminase and alkaline phosphatase activities, increase in inflammatory cytokine and bile acid concentrations, as well as altered amino acid concentrations in the blood. Interestingly, compromised blood capillary morphology coupled with astrogliosis and microgliosis were observed in brain hippocampus of the WD mice, indicating neuroinflammation or a disrupted neurovascular unit. Moreover, attention was impaired in WD-fed mice along with the observations of impaired motor activity and balance, enhanced anxiety, and stereotyped head-twitch response (HTR) behaviors. Analysis of neurotransmitters and modulators including dopamine, serotonin, GABA, glutamate, and acetylcholine showed region-specific dysregulation in the brain of the WD-fed mice. In conclusion, the induction of MASH in mice is accompanied by the alteration of cellular morphology and neurotransmitter expression in the brain, associated with compromised cognitive functions.

## 1. Introduction

Obesity is increasing worldwide [1]. One reason is the increasing consumption of the so-called “Western diet (WD)” that combines high sugar, fat, and cholesterol [2]. Besides cardiovascular and metabolic diseases [3], the brain and the liver are among the most affected organs of WD-associated obesity. Obesity is associated with several psychiatric, behavioral, and cognitive alterations, including depression, poor cognitive performance, impaired memory, and increased anxiety [4,5,6]. In the liver, overnutrition causes an increased risk of MASLD—former nomenclature: non-alcoholic fatty liver disease (NAFLD)—of which more than 20% of the population are affected worldwide [7,8]. MASLD may progress to steatohepatitis and fatal end-stage liver disease [9].

To induce MASLD in rodents, obesogenic diets have been used, such as the commercially available WD containing high sugar, unsaturated fat, and cholesterol [2]. Over a period of one year, this diet induced key features of human Metabolic Dysfunction-Associated Steatohepatitis (MASH)—former nomenclature: nonalcoholic steatohepatitis (NASH)—including the formation of lipogranulomas, fibrosis, the release of inflammatory cytokines, ammonia, and bile acids (BA) in a well-reproducible time-course [2] that can be life-threatening, as it can lead to liver cirrhosis or cancer [10]. It has been demonstrated that the peripheral inflammation observed in MASH patients may cause endothelial damage and the formation of microthrombi in the brain parenchyma, leading to neuroinflammation and subsequent neurologic impairments. Interestingly, obesity has been identified as a predictor for clinical decompensation (a functional structural or systemic deterioration) in patients with liver disease. The decompensation includes hepatic encephalopathy (HE), ascites, and variceal hemorrhage. The proportion of patients with clinical decompensation increases with a higher body mass index (BMI) occurring in 42.9% of patients with obesity, of which 31% presented HE [11]. Furthermore, a prospective study of 1773 adults with MASLD identified HE and ascites as the most common hepato-related decompensation events [12].

It is currently unclear if cognitive alterations in obesity are a direct consequence of diet ingredients, such as sugar or cholesterol, on the brain, or indirectly caused via mediators from other organs, such as inflammatory cytokines, BAs, and ammonia released from the liver into the systemic circulation [13]. To address these questions, reliable animal models that recapitulate the key symptoms of human disease via comparable mechanisms are required. In the present study, we analyzed the liver-brain axis in a mouse model of MASLD induced by feeding a Western diet for 48 weeks.

## 2. Materials and Methods

Materials used in this study are summarized in Supplementary Table S1.

*Experimental animals and feeding.* Eight-week-old male C57BL/6N mice (Janvier Labs, Le Genest-Saint-Isle, France) were used. The mice were housed individually under 12-h light/dark cycles at a controlled ambient temperature of 25 °C with free access to water. Immediately after arrival, the mice were fed *ad libitum* either with Ssniff R/M-H, 10 mm standard diet (SD) (Ssniff, Soest, Germany), or Western diet (WD) (Research Diets, New Brunswick, NJ, USA) for 48 weeks (for details see Supplementary Table S2). All experiments were approved by the local animal protection committee (LANUV, North Rhine-Westphalia, Germany, application number: 81-02.04. 2020.A304).

*Sample collection.* Blood, liver, and brain tissue samples were collected seven days after the last behavioral test. The blood samples were collected from the portal and hepatic veins, as well as from the right heart chamber, as previously described [14,15]. The liver tissue samples were collected from the left liver lobe and were fixed in 4% paraformaldehyde for two days and then processed for preparation of paraffin sections [16,17]. The brain tissue was cut into two halves along the midsagittal plane. One half was first fixed in 4% paraformaldehyde for two days, followed by incubation in 30% sucrose for two days. It was finally embedded in NEG-embedding media (Epredia, Kalamazoo, MI, USA) and stored at −80 °C until used for 2D/3D staining. The other half was used to dissect different brain areas, namely the hippocampus, cerebellum, cortex, brain stem, and striatum, and then snap-frozen in liquid nitrogen until used for neurotransmitter assays.

*Liver enzyme assay.* Transaminase (ALT and AST) and alkaline phosphatase activities were measured in undiluted cardiac whole blood using Piccolo Xpress Clinical Chemistry Analyzer (Abaxis, Union City, CA, USA) and General Chemistry 13 panel (Hitado, Möhnesee, Germany) [18].

*Cytokine assay.* Cytokine levels were measured in plasma separated from heart blood using the following commercially available kits: IL6, IL10, TNF-α (R&D Systems, Minneapolis, MN, USA), and IL-12 p40+p70 (Abcam, Cambridge, UK). The manufacturers’ protocols were followed.

*Ammonia assay.* Ammonia concentrations were measured from 20 µL of whole blood samples collected from the portal and hepatic veins and heart using a PocketChem BA PA-4140 ammonia meter (Arkray, Amstelveen, The Netherlands) [2].

*LC-MS-analysis of bile acids, amino acids, and urea.* For each type of analysis, 5 µL of plasma was supplemented with 5 µL of the respective stable-isotope labelled internal standard mix (bile acids: Chenodeoxycholate-D4, Cholate-D4, Cholic acid sulfate-D4, Deoxycholate-D4, Glycochenodeoxycholate-D4, Glycocholate-D4, Glycodeoxycholate-D4, Glycolithocholate-D4, Glycoursodeoxycholate-D4, Lithocholate-D4, β-Muricholate-D5, Taurochenodeoxycholate-D4, Taurocholate-D4, Taurocholic acid sulfate-D4, Taurodeoxycholate-D4, Taurolithocholate-D4, α-Tauro-muricholate-D4, β-Tauro-muricholate-D4, Tauroursodeoxycholate-D4, Ursodeoxycholate-D4, α-Muricholate-D5, all 500 nmol/L (Cambridge Isotope Laboratories, Tewksbury, MA, USA; Toronto Research Chemicals, North York, ON, Canada); amino acids: MSK-CAA-1, 10 µmol/L (Cambridge Isotope Laboratories, Tewksbury, MA, USA); urea: 15N2-13C-urea, 50 µmol/L (Sigma Aldrich, St. Louis, MO, USA)) and protein was precipitated by the addition of 40 µL ice-cold methanol. After centrifugation at 21,000× *g* for 5 min at 4 °C, the supernatant was transferred to a sample vial.

For analysis of bile acids, 5 µL was injected to the LC-MS-system. Chromatographic separation was facilitated by a Vanquish Horizon UHPLC (ThermoFisher Scientific, Waltham, MA, USA) equipped with a Waters Acquity UPLC Peptide CSH C18 column (1 mm I.D. × 150 mm length, 130 Å, 1.7 µm particle size) and an Acquity UPLC Peptide CSH C18, VanGuard Pre-Column (2.1 mm I.D. × 5 mm, 130 Å, 1.7 µm) using a flow rate of 150 µL/min at 60 °C. The binary gradient (solvent A: 0.05% formic acid, 3 mmol/L NH4COOH; solvent B: acetonitrile) was as follows: from 25–50% B in 15 min, 50–90% B in 1 min, kept at 90% B for 4 min, back to 25% in 1 min, re-equilibration for 4 min. The QExactive mass spectrometer operated in negative Full-MS-mode (*m*/*z*-range 370–600; resolution 140,000; AGC 1 × 10^6^) from 0–19 min and in positive t-SIM-mode (target 401.3414 *m*/*z* (7α-Hydroxy-4-cholesten-3-one); isolation window 3 *m*/*z*; resolution 140,000; AGC 1 × 10^5^) from 19–25 min. The analysis of amino acids was accomplished by injection of 1 µL sample to the same LC-MS-system equipped with a MN NucleoShell Bluebird RP18 (3 mm I.D. × 150 mm length, 90 Å, 2.7 µm particle size) using 400 µL/min flow rate and the following binary gradient (solvent A: 0.1% formic acid, 0.02% heptafluorobutyric acid; solvent B: 0.1% formic acid in acetonitrile): 2% B for 2.5 min, 2–50% B in 3.75 min, 50–90% B in 0.25 min, 90% B for 2.25 min, 90–2% in 0.25 min, and re-equilibration at 2% B for 3 min. The QExactive mass spectrometer operated in positive mode acquiring, alternatingly, Full-MS and AIF spectra at a resolution of 140,000 and AGC 1 × 10^6^; the *m*/*z*-range was 70–300 for Full-MS and 50–300 for AIF spectra. Urea analysis was done with 2 µL sample volume on the same LC-settings as for the amino acid analyses, but the mass spectrometer was operated in tSIM-mode with an isolation width of 1 *m*/*z*, 140,000 resolution, AGC 1 × 10^5^. Quantification was accomplished using Skyline Daily (v22.2.1.351) [19]. Data were normalized to the respective stable-isotope-labelled standards or -in case of missing standards (ω-Muricholate and 7α-Hydroxy-4-cholesten-3-one)- to α-Muricholate-D5. Calibration curves ranged from 1–10,000 nmol/L for bile acids, 1–100 µmol/L for amino acids and 10–1500 µmol/l for urea. All data are available from the Panorama website (https://panoramaweb.org/project/Panorama%20Public/begin.view (search term: author: Custodio)).

*Histopathology and immunohistochemistry analyses of the liver.* Hematoxylin and eosin (H&E), Sirius red, and immunohistochemistry analyses were performed in 4 μm-thick, 4% PFA-fixed paraffin-embedded liver tissue sections. H&E and immunohistochemistry were performed using the Discovery Ultra Automated Slide Preparation System (Roche, Mannheim, Germany) as previously described [20,21]. Antibodies used in immunohistochemistry are listed in Supplementary Table S3. For Sirius red staining, a commercially available kit (Polysciences Inc., Warrington, PA, USA) was used according to the manufacturer’s protocol [22]. A whole slide scanning of stained slides was performed using a digital scanner (AxioScan.Z1; Zeiss, Jena, Germany).

*Immunohistochemistry analysis of the brain.* Five and 100 µm thick cryosections were prepared. The 5 μm-thick sections were stained using antibodies against serotonin in the Discovery Ultra Automated Slide Preparation System (Roche, Mannheim, Germany) (Supplementary Table S3). The 100 μm-thick slices were stained manually using antibodies against GFAP (astrocytes marker), Iba1 (microglia marker), and CD31 (endothelial cell marker); the nuclei were counterstained using Hoechst (dilution 1:5000) (Supplementary Table S3). Three-dimensional z-stacks were performed using a confocal scanner (Celldiscoverer 7, Zeiss, Jena, Germany). Reconstructions and quantifications were performed using Arivis Vision4D 4.0 software (Zeiss, Jena, Germany). Each signal was subjected to a customized pipeline. Morphology and denoising filters were applied to enhance the signal from bright objects and to homogenize the background. Threshold-based segmentation of the CD31 signal was applied in predefined 3D regions to process and quantify the total volume and the surface area of blood vessels. Neural tracing of GFAP and Iba1 signals were applied in the same 3D regions to analyze and quantify the total volume and surface area of astrocytes and microglia. Image analysis parameters were kept constant for all 3D stacks.

*Arginase1 and glutamine synthetase staining quantifications.* The area of arginase1 and glutamine synthetase were segmented and quantified using a specialized whole slide image analysis software as previously described [2].

*Neurotransmitter quantification.* Neurotransmitter analysis was conducted as previously published [23]. Due to a different matrix, the extraction protocol was adjusted: The extraction buffer was prepared as described, but the concentration of deuterated standards was increased to 500 nM. Additionally, L-glutamic-2,3,3,4,4-d5 acid (Sigma-Aldrich, St. Louis, MO, USA) was added. Samples were added with 200 µL (hippocampus, cerebellum, striatum), 500 µL (brainstem), or 1000 µL (cortex) buffer and homogenized by 4× freeze-thaw cycles (1 min 37 °C, 1 min liquid nitrogen) and by sonification (5 × 20 s, amplitude 100, cycle 1). Samples were centrifuged (16,060 g, +4 °C), transferred to Spin-X^®^ Centrifuge Tube Filter 0.22 µm (Corning Inc., Corning, NY, USA), and finally transferred to vials for LC-MS/MS analysis. The data were normalized to protein levels which were measured using bicinchoninic acid (BCA) assay protocol.

*Behavioral analyses.* The sequence of behavioral tests was carried out from the least to the most stressful, minimizing the carryover effect of the preceding tests while taking into consideration the tests that needed habituation or training allowing mice to adapt to the platform. Thus, removing novelty and biased effects which may produce confounding effects in the analyses of the animal behaviors. The sequence is as follows.

*Open-field test (OFT):* The test evaluated the locomotor activity (i.e., distance moved (cm), movement duration (s)) of mice using a previous protocol [24]. Mouse locomotor activity was assessed in a square gray Plexiglas container with an open-field (OF) arena (40 × 40 × 40 cm^3^; code: 47432; Ugo Basile, Varese, Italy) and evaluated using a video tracking software, Noldus Ethovison XT v17.5 (RRID: SCR_000441; Wageningen, Netherlands). Mice were gently placed in the center of the open-field arena and allowed to explore for 10 min for three consecutive days. The first two days served as a habituation period that eliminated the bias of novelty in the “open field”. Moreover, aside from the motor activity parameters measured, the center- and periphery-field explorations were assessed as a possible measure of anxiety in mice.

*Rotarod (RT) test:* Maintaining motor balance and coordination were assessed using a rotating rod (rotarod; RR) (code: 47650; Ugo Basile, Varese, Italy) at 36 rotations per minute (rpm) based on a previous study [25]. Like the OFT, this experiment was also conducted for three consecutive days in which the first two days served as the habituation period while the last day served as the actual test. Mice were exposed to the rotating rod for 10 min. The latency to the first fall and falling frequencies were recorded.

*Elevated-plus maze test (EPMT):* Mice were exposed to the elevated plus maze (EPM) to evaluate their anxiety-like behaviors using a previous protocol [26]. The plus-maze consisted of four arms: two open arms (80 × 5 cm^2^) and two closed arms (80 × 5 cm^2^) enclosed by 35-cm-high walls with a delimited central area of 5 × 5 cm^2^ (code: 40143; Ugo Basile, Varese, Italy). The entire maze was elevated to 60 cm above the floor. Indirect lighting was utilized to prevent the formation of hard shadows, which can be an area of preference for mice. At the start of the test, each mouse was placed at the center of the maze facing one of the open arms. Noldus Ethovison XT v17.5 (RRID: SCR_000441; Wageningen, Netherlands) identifies an entry into an arm when the mouse places all four paws over the invisible line marking that area. The number of entries and the time spent in the open arms were used to identify the percentage of open arm entries using a formula (100 × open/total entries).

*Cliff avoidance test (CAT):* The CAT evaluated impulsive behaviors in mice based on a previous study [27]. A round wooden platform (diameter 20 cm; thickness 1 cm), supported by a cylindrical-shaped PVC rod (height 50 cm), was used. During the test, each mouse was placed at the center of the platform and observed for 10 min. The latency of the first fall (the time the subject fell from the platform) and falling frequencies (number of falls) were recorded using the Noldus Ethovison XT v17.5 video tracking software (RRID: SCR_000441; Wageningen, The Netherlands).

*Head-twitch response test (HTRT):* The head-twitch responses (HTR) in mice test was performed as previously described [28]. The behaviors were recorded for 10 min, following 2 min of habituation. Each mouse was placed in the center of a transparent acrylic cage of a multi-purpose ActiMot2 systems unit measuring 250 × 250 mm with a 200 mm height (TSE systems, Chesterfield, MO, USA) and allowed to explore. Video recording was then extracted, and the number of HTRs of each mouse was analyzed. Moreover, the number of jumping behaviors of mice was analyzed from the same video recording.

*Marble burying test (MBT)* examines repeated and compulsive-like behaviors in mice. The test was evaluated in an empty cage with 20 arranged glass marbles (20 mm diameter) evenly spaced (4 × 5 arrangements) in a 6 cm depth of corncob bedding. Mice were first placed in a cage without marbles for 10 min, then transferred to the test cage (with marbles) for another 10 min, and then carefully removed and returned to their home cage based on a previous study [29]. The number of marbles buried in the corncob bedding with more than 50% of the surface area was counted after each trial.

*Novel object recognition test (NORT):* This cognitive test is a standard tool for measuring rodent cognitive processes (i.e., recognition memory, attention). The test consists of three phases: habituation, familiarization, and discrimination following a previously published study [30]. Each mouse was placed in an open-field box for 10 min/day for two consecutive days (habituation phase) to remove the biased effect of novelty towards the platform. Following the last day of habituation, two identical objects (wooden boxes, A1 and A2) were placed in opposite and equidistant locations, and each mouse was allowed to explore and familiarize themself with the objects for 10 min (familiarization phase). The mice were then returned to their home cages and allowed to stay for 30 min before being reintroduced into the arena for the ‘discrimination phase.’ In this phase, objects A1 and A2 are replaced by objects identical to the familiar object (A3) and a novel object (B). Explorations and visitations on all objects present during the familiarization and discrimination phases were recorded using Noldus Ethovison XT v17.5 (RRID: SCR_000441; Wageningen, The Netherlands). The total investigation time (A1 + A2) was determined in the familiarization phase, and the discrimination index (B − A3/B + A3) during the discrimination phase. The discrimination index ranges between +1 and −1: a positive score indicates more time spent with the novel object, a negative score indicates more time spent with the familiar object, and zero (0) indicates a null preference.

*Object-based attention test (OBAT):* Another validated tool to measure attention in mice is the OBAT. Each mouse was habituated in two empty open-field boxes (exploration chamber [EC] and test chamber [TC]) for 10 min/day for two consecutive days, based on a previous study [30]. Correspondingly, each mouse was placed in the EC containing five objects of the same size but different shapes and colors during acquisition. The five objects were of the following shapes: cube, half-cylinder, cuboid, pyramid, and cylinder. Next, each mouse was placed in the TC containing a familiar (cylinder) and a novel (rectangular prism) object, allowing another 10 min of object exploration. The positions for each object in the EC and TC were fixed throughout the study. The following formula [TN/(TF + TN) × 100] was used to calculate the recognition index in mice, where TF (familiar test object) and TN (novel test object) are the times spent exploring the objects in the TC. Furthermore, the “preference index” was calculated using the formula TN/TF, where time spent exploring the novel object is divided into the time spent in the familiar object. The activity of mice was recorded using video tracking software Noldus Ethovison XT v17.5 (RRID: SCR_000441; Wageningen, The Netherlands).

*Y-maze test (YMT):* Cognition of the mice was evaluated through spatial learning and memory. Each mouse was placed in one of the Y-maze (45 × 10 × 20 cm) arms and allowed to explore freely for 10 min based on a previous study [31]. The study defines an “arm entry” as the entry of all four paws (mice) into an arm and the “alternation behavior” (actual alternations) as a consecutive entry into three arms. Visits to three arms and alternations were measured using the Noldus Ethovison XT v17.5 video tracking software (RRID: SCR_000441; Wageningen, Netherlands). The percentage of spontaneous alternation was calculated as the ratio of actual alternations to the maximum number of alternations (total number of arm entries minus two) multiplied by 100 (% alternation  =  [(number of alternations)/(total arm entries − 2)] × 100).

*Barnes maze test (BMT):* The BMT is a dry-land-based rodent behavioral paradigm for assessing spatial learning and memory in mice based on a previous study [30]. The circular gray maze has a diameter of 92 cm and a height of 60 cm (code: 40193; Ugo Basile, Italy). It is made with steel with an anti-slip and “warm” surface texture selected for rodent comfort. In the circular maze there are 20 equally spaced holes (∅: 5 cm) located 3 cm from the border. In this open environment, mice naturally seek a dark, enclosed surrounding place provided by a black goal box (6 × 12 × 6 cm) or a shelter chamber equipped with a ramp attached magnetically beneath one of the holes. Visual cues are placed around the maze, which act as spatial cues. Each trial began by placing the mouse in a black starting cylinder (∅: 8 cm, height: 12.5 cm) at the center of the platform, which was removed after 30 s, allowing mice to explore the maze freely. Mice were habituated to the platform two days before the acquisition trials (unrecorded) to remove the bias of novelty. The spatial acquisition was organized in 2 daily trials from days 1–4. Each trial consisted of two 3 min trials, with 30 min inter-trial intervals during which animals could return to their home cage for rest. A mouse that failed to find the target box within 3 min was gently guided to the location of the goal box. For a failed mouse, 180 s was recorded as the escape latency. All animals remained in the goal box for 60 s after entry. This study defines ‘latency’ as the time the mouse enters the goal box. Errors were identified as the number of visits to other holes that do not contain the goal box. Repeated errors were the number of repeated visits to other holes not containing the goal box. On Day 5, a probe trial (180 s) was conducted, during which the goal box was removed and replaced with a false shelter mimicking the color of the goal box. The mouse was allowed to explore the maze and visit the target hole. The adjacent holes were divided into four imaginary quadrants (e.g., target, positive, negative, and opposite). The distribution of visits among all holes and the time spent in each quadrant were recorded. All movements and explorations for each mouse were recorded using video tracking software Noldus Ethovison XT v17.5 (RRID: SCR_000441; Wageningen, The Netherlands).

*Statistical analysis.* Data were analyzed using Prism software version 9.5.1 (RRID:SCR_002798; GraphPad Software, Inc., La Jolla, CA, USA). In all available figures, the animal numbers and recorded data points were indicated. Results were analyzed using either an unpaired *t*-test (two-tailed), Mann-Whitney U test, or two-way analysis of variance (ANOVA), followed by Tukey’s multiple comparisons tests. A level of probability of *p*  ≤  0.05 was defined as the threshold for statistical significance.

## 3. Results

### 3.1. Long-Term Feeding on Western Diet Induces Steatohepatitis and Compromises Blood Biochemistry in Mice

**Figure 1:**
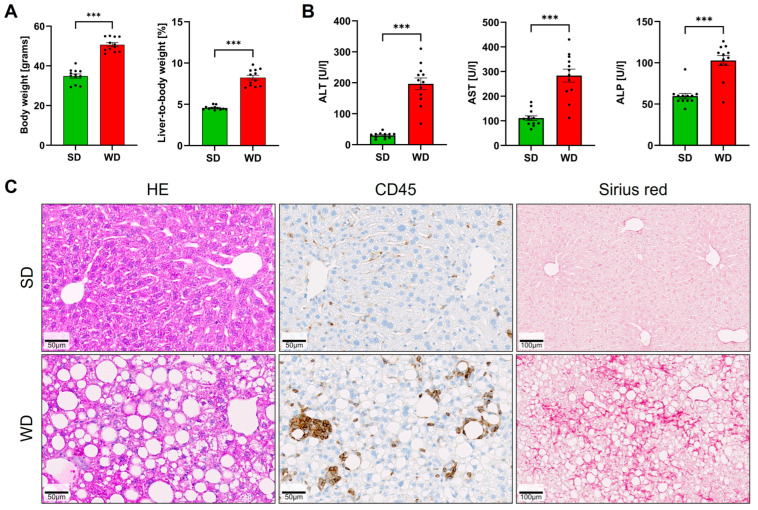
Induction of steatohepatitis by 48 weeks of Western diet (WD) and standard diet (SD). (**A**) Body weight and liver-to-body-weight ratio. (**B**) Liver enzyme activities of alanine aminotransferase (ALT), aspartate aminotransferase (AST), and alkaline phosphatase (ALP) in plasma. (**C**) Hematoxylin-eosin (HE) staining, immunostaining with antibodies directed against the pan-leukocyte marker CD45, and visualization of fibrosis by Sirius red. Data are presented as the mean ± SE of 12 mice per test. Mann-Whitney U; ***: *p*  <  0.001.

To induce steatohepatitis, mice were fed a Western diet for 48 weeks. Compared to the standard diet-fed controls, the mice on WD showed a significantly increased body weight and liver-to-body-weight ratio (Figure 1A). Liver enzyme activities of ALT, AST, and ALP in plasma were increased, indicating a significant level of liver damage (Figure 1B). Hematoxylin and eosin staining visualized large lipid droplets in the WD-fed mice (Figure 1C). Immunostaining with the pan-leukocyte marker CD45 demonstrated the infiltration of immune cells that particularly surrounded large lipid droplets forming lipogranulomas (Figure 1C). Moreover, the WD-fed mice developed diffuse fibrosis as evidenced by Sirius red staining (Figure 1C).

**Figure 2:**
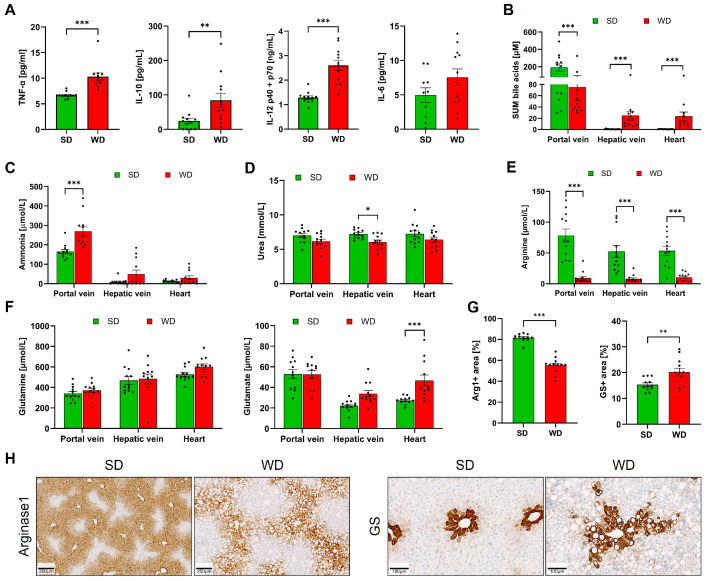
Clinical chemistry and immunostaining of arginase1 and glutamine synthetase in mice after 48 weeks of Western diet (WD) and standard diet (SD). (**A**) TNF-α, IL-10, IL-12, and IL-6 in plasma. (**B**) Sum bile acids (BA) from plasma taken from the portal vein, hepatic vein, and left heart chamber. (**C**) Ammonia (plasma). (**D**) Urea (plasma). (**E**) Arginine (plasma). (**F**). Glutamine and glutamate (plasma). (**G**) Quantification of arginase1 and glutamine synthetase immunostainings from whole slide scans. (**H**). Representative immunostainings for arginase1 (periportal zonation) and glutamine synthetase (GS) (pericentral zonation). Data are presented as the mean ± SE of 12 mice per test. (**A**) Mann-Whitney U, (**B**–**H**) two-way ANOVA with Tukey’s multiple comparisons; *: *p* ≤ 0.05, **: *p*  <  0.01; ***: *p*  <  0.001.

Plasma analysis of inflammation markers demonstrated a significant increase in TNF-α, IL-10, and IL-12. In contrast, a moderate increase of IL-6 did not amount to statistical significance (Figure 2A). In WD-fed mice, plasma BA concentrations were decreased in the portal vein (representing the liver inflow). In contrast, concentrations in the hepatic vein (the liver outflow) were significantly increased (Figure 2B), suggesting that the ability of the liver to clear BA from the blood was reduced in steatohepatitis. Corresponding to the increased concentrations in the hepatic vein, BA concentrations were also higher in the systemic circulation of the WD mice, as evidenced by the analysis of plasma from the left heart chamber (Figure 2B). Ammonia concentrations were significantly increased in the portal blood of the WD mice, but no significant changes could be detected in the hepatic vein or systemic blood (Figure 2C). Analysis of urea showed a modest decrease in the WD-fed mice that amounted to statistical significance only in the hepatic vein blood (Figure 2D). Concentrations of arginine in the portal vein, hepatic vein, and in heart blood were strongly reduced in mice fed with WD compared to a standard diet (Figure 2E). In contrast, no significant differences were obtained for plasma glutamine (Figure 2F). Glutamate was significantly higher in the systemic blood of the WD-fed mice (Figure 2F). Since the expression of arginase1 and glutamine synthetase (GS) has been reported to be influenced by the WD [2], both enzymes were investigated by immunostaining of formalin-fixed paraffin-embedded tissue (Figure 2G,H). The area of arginase-positive immunostaining was strongly reduced in WD-fed mice, while the area of positive GS was increased. Together, the results show that long-term feeding of a Western diet induces steatohepatitis and alters blood biochemistry.

### 3.2. Induction of Steatohepatitis by Western Diet Coincides with Impaired Behaviors in Mice

**Figure 3:**
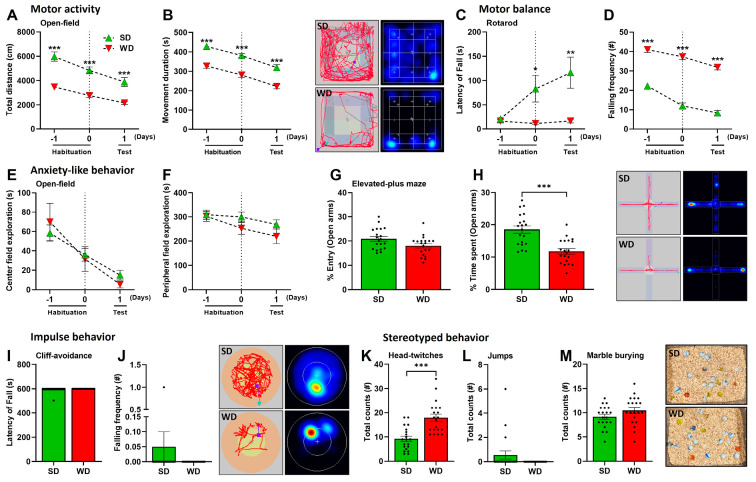
Motor, anxiety, impulsivity, and stereotypical behaviors of mice following 48 weeks of Western diet (WD) and standard diet (SD). (**A**,**B**) Total distance (cm) and movement duration (s) during the Open-field test (OFT). (**C**,**D**) Latency of fall (s) and falling frequency (#) in the Rotarod test (RT). (**E**,**F**) The center and peripheral field explorations (s) in the OFT. (**G**,**H**) Entry and time spent (%) in the open arms during the Elevated-plus maze test (EPMT). (**I**,**J**) Latency of fall (s) and falling frequency (#) in the Cliff-avoidance test (CAT). (**K**) Total counts of Head-twitch responses (HTR). (**L**) Total counts of jumps. (**M**) Total counts of buried marbles during the Marble-burying test (MBT). Data are presented as the mean ± SE of 20 mice per test. (**A**–**F**) two-way ANOVA with Tukey’s multiple comparisons, (**G**–**M**) two-tailed unpaired *t*-test. *: *p* ≤ 0.05, **: *p*  <  0.01; ***: *p*  <  0.001. Representative tracks, heatmaps, and photographs (marble burying) are shown.

To investigate the influence of WD/steatohepatitis on cognitive functions of the brain, mice were exposed to various behavioral tests evaluating motor behavior, anxiety, impulsivity, stereotypical behaviors, and cognitive abilities. Compared with SD-fed controls, the WD-fed mice showed impairments in motor behavior as evidenced by reduced motor activity (total distance traveled), movement duration during OFT (Figure 3A,B), and impaired motor balance (shorter latency and increased falling frequency) in the RT (Figure 3C,D). Using the OFT as a measure of anxiety, we failed to identify significant changes in the center and peripheral field exploration (Figure 3E,F) between the groups. However, in the EPMT, another test measuring anxiety, we found that WD-fed mice have a slightly reduced entry (%) into the open arms (Figure 3G,H), which could be explained by the general hypoactivity in the WD mice. Moreover, these mice spent significantly shorter time (%) in the open arms of EPM, suggesting signs of anxiety. The behavior of WD-fed mice during the cliff-avoidance test (CAT), measuring the latency of fall and falling frequencies, did not differ from that of the SD-fed mice, showing their absence of impulsive behavior (Figure 3I,J). Interestingly, when evaluated for presence of stereotyped or repetitive behaviors, we found that WD feeding induced a higher number of head twitch responses (HTR) in mice relative to the SD group (Figure 3K), but with the absence of excess jumping (Figure 3L) and marble-burying (Figure 3M).

**Figure 4:**
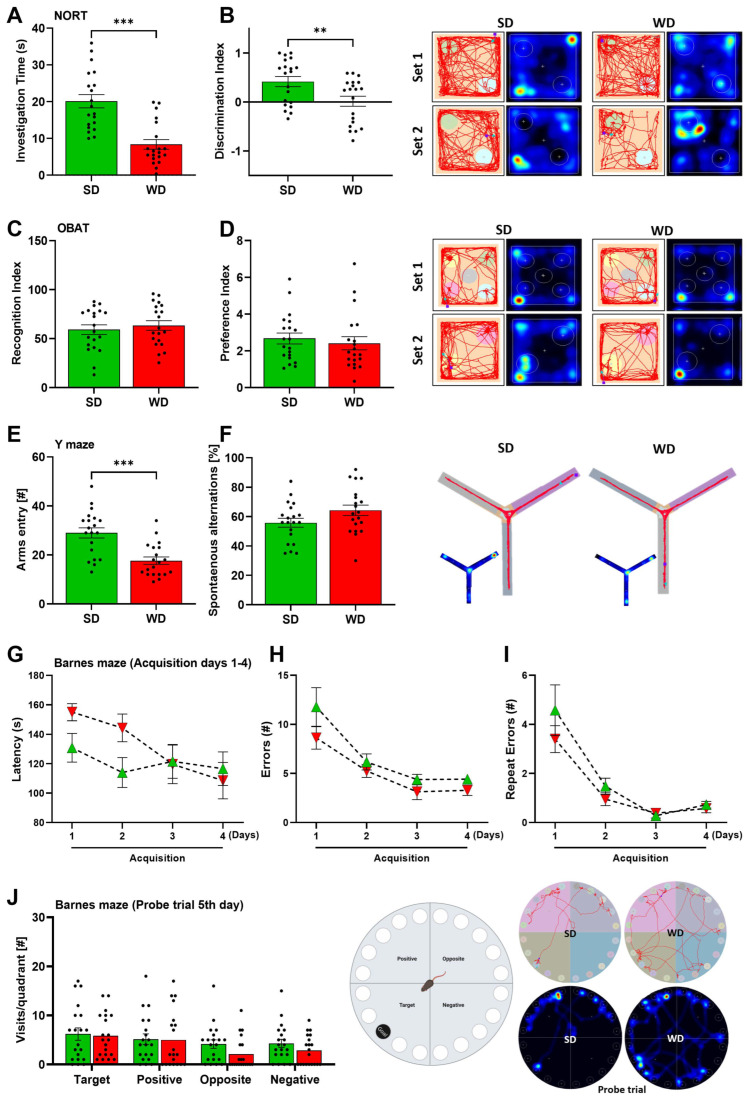
Cognitive behaviors of mice after 48 weeks of Western diet (WD) and standard diet (SD). (**A**,**B**) Investigation time (s) and discrimination index in the Novel-object recognition test (NORT). (**C**,**D**) Recognition and preference indices in the Object-based recognition test (OBAT). (**E**,**F**) Arms entry (#) and spontaneous alternations (%) in the Y-maze test (YMT). (**G**–**I**) Latency (s), errors, and repeat errors (#) during the acquisition phase of the Barnes maze test (BMT). (**J**) Visits per quadrant during the Probe trial fifth day of BMT. Data are presented as the mean ± SE of 20 mice per test. Compared to SD group, (**A**–**F**) two-tailed unpaired *t*-test, (**D**,**E**) two-way ANOVA with Tukey’s multiple comparisons. **: *p*  <  0.01; ***: *p*  <  0.001. Representative tracks and heatmaps per experiment are shown.

Interestingly, we found that WD-fed mice had lower investigation time and discrimination index in the NORT (Figure 4A,B), indicating impairments in object recognition, a complex cognitive function of attention. However, OBAT results did not support this finding, since recognition and preference indices did not vary with SD-fed mice (Figure 4C,D). During the YMT, we found that the amount of arms entry in WD-fed mice was significantly lower compared with SD-fed mice (Figure 4E), which may be supported by the general hypoactivity in these mice. However, no significant change in the spontaneous alternations were observed in WD-fed mice (Figure 4F). Furthermore, when exposed to the BMT, the WD-fed mice showed similar behavior to SD-fed mice. During the first two days of the acquisition phases, a slight increase in the latency to locate the “goal box” was observed in WD-fed mice but was insignificant compared to SD-fed mice (Figure 4G). Interestingly, during the last two acquisition days (days 3 and 4), both groups performed similar behavior, showing that the WD-fed mice progressively improved in locating the goal box. Moreover, the number of errors and repeated errors were the same for both groups throughout the four days of the acquisition phase errors (Figure 4H,I) and, more importantly, when exposed on the last day (probe trial 5th day), which measured the short-term memory in mice, visitations towards the ‘target’ quadrant were the same for both groups (Figure 4J). Given the findings observed with YMT and BMT, 48 weeks of WD feeding did not induce memory impairments in mice. Overall, WD induces motor impairments, anxiety, HTR, and cognitive impairments (in object recognition) in mice without significant changes in other behaviors.

### 3.3. Region-Specific Alterations of Neurotransmitter Levels in the Brain of Western Diet-Fed Mice

**Figure 5:**
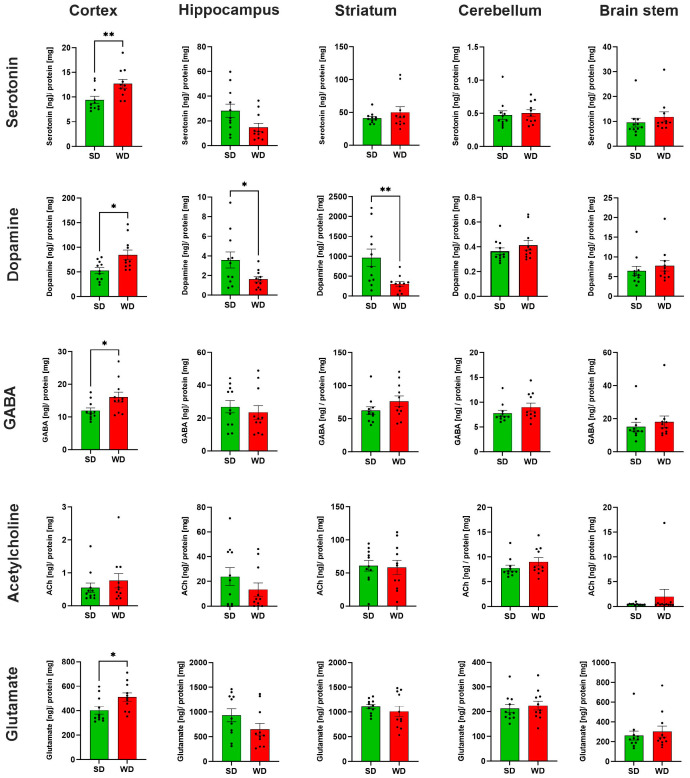
Neurotransmitter levels in specific brain regions of mice 48 weeks after Western diet (WD) and standard diet (SD). Data are presented as mean ± SE of 11 mice. Mann-Whitney U; *: *p* ≤ 0.05, **: *p*  <  0.01.

**Figure 6:**
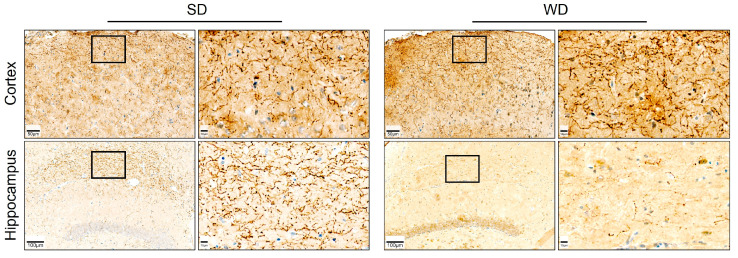
Immunostaining for serotonin on tissue sections of the hippocampus and cortex of the brain of mice 48 weeks after Western diet (WD) and standard diet (SD).

The levels of five neurotransmitters/-modulators, namely serotonin (5-HT), dopamine (DA), gamma-aminobutyric acid (GABA), acetylcholine (ACh), and glutamate (Glu), were analyzed in homogenate obtained from five specific brain regions (brain stem, striatum, cortex, hippocampus, cerebellum) (Figure 5). With respect to the different brain regions, the strongest differences were observed in the cortex. Except for Ach, all protein-normalized neurotransmitter levels were significantly increased in WD. For 5-HT, the median increased from 8.4 to 12.4 ng/mg protein; for dopamine, the respective concentrations were 51.5 and 71.7 ng/mg protein; in SD mice, average GABA levels were 11.4 ng/mg protein; while in WD, levels were 15.0 ng/mg protein; and for Glu, median values of 346.1 and 503.0 ng/mg protein caused these significant differences. Thus, on average, the neurotransmitter levels in the cortex showed a 1.4-fold increase in WD. Regarding the five neurotransmitters/-modulators, the strongest effects were observed for DA. In contrast to the cortex, this neuromodulator was reduced in the hippocampus and striatum in WD. On average, the dopamine concentrations in WD mice were reduced to 45.3% of the SD group. Even though not reaching statistical significance (*p* = 0.06), the 5-HT levels in the hippocampus of the WD mice were reduced. These divergent and relatively large effects for 5-HT encouraged us to perform immunostaining for serotonin in hippocampal and cortex sections. A clear decrease of the 5-HT signal in the WD compared to SD-fed mice was obtained for the hippocampus, while the opposite was observed in cortex samples (Figure 6). In summary, WD non-selectively increased neurotransmitter/-modulator levels in the cortex and simultaneously reduced dopamine levels in the hippocampus and striatum. In other brain areas, no differences could be observed.

### 3.4. Compromised Blood Capillaries, Astrogliosis, and Microgliosis in the Brain of Western Diet-Fed Mice

**Figure 7:**
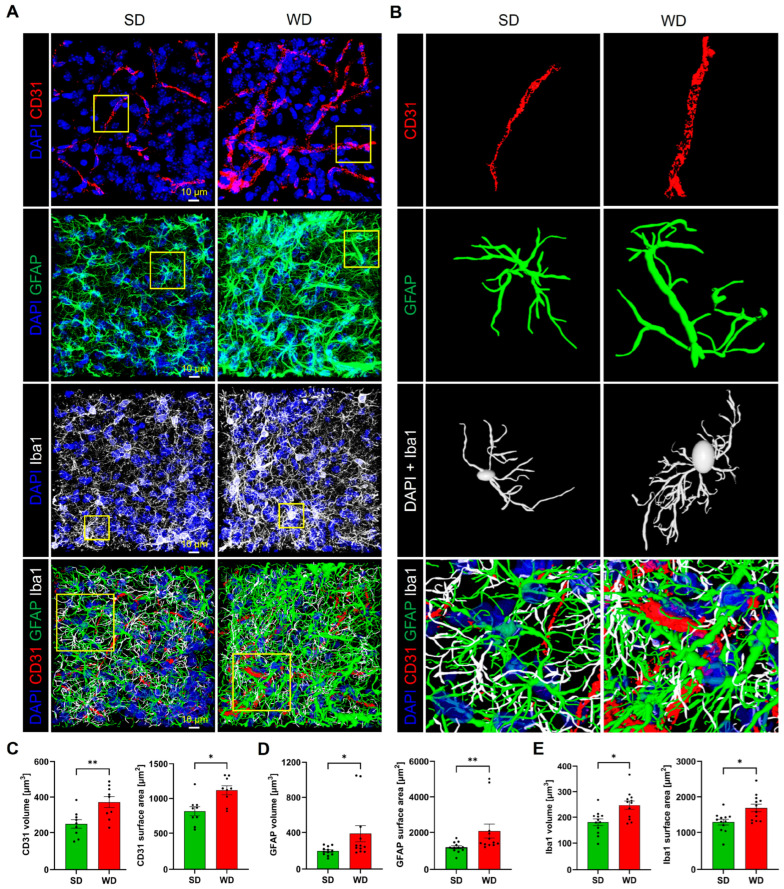
Histological alterations of hippocampal tissue after 48 weeks of Western diet (WD). (**A**) Immunostaining for the endothelial marker CD31, the astrocyte marker GFAP, and the microglia marker lba1. DAPI visualizes nuclei. (**B**) 3D reconstructed individual structures: microvessel segments (from the area indicated in (**A**)), astrocyte, microglial cell, and the intertwined network of all three structures. (**C**–**E**) Quantifications of microvessels, astrocytes, and microglia. Data are presented as mean ± SE of 12 mice. Mann-Whitney U; *: *p* ≤ 0.05, **: *p*  <  0.01.

Since several relevant alterations observed so far were obtained for the hippocampus, we focused on this brain region for histological analysis. For this purpose, microvessels were visualized by the endothelial cell marker CD31, while astrocytes and microglia were stained by antibodies directed against GFAP and lba1, respectively. A striking feature observed by CD31 immunostaining was the dilatation of microvessels (Figure 7A). Quantification of the CD31 signal showed increased volume and surface area of blood vessels in the WD compared to SD-fed mice (Figure 7A–C).

Immunostaining of astrocytes showed a higher fraction of GFAP-positive structures in the WD compared to control mice (Figure 7A,B,D). Upon reconstruction of individual astrocytes (Figure 7B), this increase seems to be due to an increased number of branches and a higher diameter of the individual branches. Similarly, the total area of lba1-positive regions representing microglia was significantly increased in the WD-fed mice (Figure 7A,B,E). Inspection of individual reconstructed microglial cells (Figure 7B) demonstrates the wider, more extensively branched network of cell protrusions and the larger cell body in the mice fed with WD, indicating activation of this cell type.

## 4. Discussion

Significant epidemiological evidence has provided information that a diet high in saturated fat and simple carbohydrates induces metabolic disorders leading to behavioral impairments, specifically cognitive dysfunctions [32,33]. This study sought to understand how a Western diet (WD) containing high fructose, saturated fat, and cholesterol previously rendered key features of human MASH in mice [2] able to induce behavioral impairments by assessing brain biochemical and physiological changes. Findings show that MASH in mice resulting from 48 weeks of WD feeding alters neurotransmitter levels in the cortex (5-HT, DA, GABA, and Glu), striatum (DA), and hippocampus (DA, 5-HT), brain regions important in motor behavior, cognitive functions, and anxiety. Moreover, microvessel morphology was altered and astrocytes as well as microglia were activated. All these findings may contribute to the behavioral impairments induced by the WD-induced MASH.

Using a previously established protocol for the induction of steatohepatitis by long-term (48 weeks) consumption of WD [2], we observed that the liver phenotype reported in this study was reproducible. Given that the liver was already severely damaged, as evidenced by the global increase in liver enzymes (e.g., ALT, AST) and plasma ALP, it is likely that liver functions were compromised. The WD-induced steatohepatitis increased systemic (plasma) inflammatory cytokines, such as TNF-α, IL-10, and IL-12. The TNF-α and IL-12 are pro-inflammatory cytokines and mediators, whereas IL-10 is an anti-inflammatory cytokine, suggesting that simultaneous activation of some pro- and anti-inflammatory mediators occurs during the inflammatory responses in WD-fed mice, previously identified in inflammatory diseases as a form of mandatory immune response [34,35]. Moreover, plasma ammonia and BAs were increased along with dysregulations of ammonia by-products (i.e., urea) and amino acids (i.e., arginine, glutamate) involved in ammonia production. Herein, we found increased ammonia levels, a known potent neurotoxin found to induce psychological and behavioral disturbances at preclinical and clinical levels [36,37]. In cases of liver damage, as with the steatohepatitis present in WD-fed mice, ammonia could build up in the blood, reach the brain, and impair brain functioning [38,39]. However, for the present mouse model, this interpretation should be treated with caution, since significant elevations of ammonia were only observed in the portal vein, but not in the hepatic vein and the heart blood (though an increased trend was observed). Apart from ammonia, BAs represent a further candidate that may be responsible for impaired cognitive functions. Unlike ammonia, plasma BAs were reduced in the portal vein but increased in the hepatic vein and heart blood. The elevated plasma BAs in the hepatic vein and particularly in heart blood indicate increased systemic BA circulation which could reach the brain. Serum BAs are significantly increased after liver damage, which may possibly disrupt the blood-brain barrier (BBB) and cause brain damage [40]. Interestingly, mice exposed to streptozotocin-high-fat diet (STZ-HFD) for 16 weeks developed MASH and showed elevated blood ammonia and BA concentrations which resulted in enhanced anxiety in mice [40]. Overall, this shows that WD-induced steatohepatitis induces dysregulation of the homeostasis of both ammonia and BA, which could impact brain and behavior functions.

It is becoming increasingly evident that peripheral organ-centered inflammatory diseases, including liver diseases such as MASLD/MASH, are linked with brain dysfunctions, including impairments in neurotransmission, leading to behavioral alterations [10,41,42], or in severe cases, the development of neuropsychiatric syndrome of HE [43]. Presently, WD-fed mice were inattentive and had pronounced motor impairments, enhanced anxiety and HTR behaviors. Obesity in mice may impact their performance in these behavioral tasks, particularly the observed motor impairments, which may occur independently from steatohepatitis. Moreover, WD-fed mice showed anxiety in EPMT, but not in the OFT. Both the OFT and EPMT measure anxiety in rodents [44], but different patterns of anxiety were observed between the center field exploration (OFT) and the time-spent in the open arms (EPMT), indicating that EPMT creates a stronger anxiogenic environment than OFT [45]. Additionally, WD-fed mice showed impaired cognitive attention (NORT); however, this was not replicated by the OBAT results. Thus, it could be that NORT with two objects was more sensitive than the OBAT with five objects. The NORT can also generate anxiety known as neophobia [46]. However, it should be considered that this anxiety is not related to arena novelty exploration, since mice were habituated before the test. Rather, this may be caused by the novel objects, particularly those that are almost indistinguishable from the familiar ones. Also, the low investigation time in WD-fed mice shows their unwillingness to explore objects that are difficult to discriminate. Furthermore, WD-fed mice were devoid of other behavioral impairments, showing that some aspects of behavior and cognition remain intact even with long-term WD exposure.

In the CNS, microglia, and astrocytes regulate inflammatory responses, maintain BBB homeostasis, and preserve brain immune function [47,48]. Pathologically, microglia and astrocytes become activated, along with systemic inflammatory cytokines, leading to severe inflammation. Herein, we found a compromised hippocampal capillary morphology and reactive astrogliosis and microgliosis. Previous studies have shown that neuroinflammatory responses occur already after much shorter feeding periods as in the present study. Mice fed a WD for 8 months and a lard-based HFD for 6 months were reported to show increased GFAP and Iba1 immunoreactivity [49,50]. On the other hand, short-term HFD feeding in mice for 7 days did not enhance GFAP and Iba1 protein expressions; however, after 21 days, these markers were already activated relative to chow diet [51].

It has been shown that neuroinflammation affects neurotransmission by altering membrane expression of neurotransmitters. Presently, in the cortex, all neurotransmitters, or neuromodulators, except for Ach, were significantly increased. WD-fed mice have low striatal and hippocampal DA levels. DA is both an excitatory and an inhibitory neurotransmitter and neuromodulator involved in learning, cognition, and motivation/reward systems [52]. Within the striatal neurons, DA increases the direct pathway actions via D1-like receptors, inducing excitatory effects while reducing the influence of the indirect pathway (D2-like receptors), thereby inducing inhibitory effects [53]. Moreover, hippocampal DA facilitates the encoding of salience (attentional mechanisms) and the formation of associative learning. Upon release, DA binds to D1-like receptors, promoting attention, spatial learning, episodic memory formation, and even synaptic plasticity [54]. When these brain regions are DA-deficient, slow, uncoordinated movements and impaired cognition were reported, akin to the OFT and NORT results in WD-fed mice. These results further support that low brain DA concentrations are associated with motor depression and cognitive dysfunction in MASH [55,56,57,58].

To our knowledge, increased HTR in WD-fed mice has not yet been reported. This phenomenon should be discussed in the context of increased 5-HT in the cortex. The HTR was first reported in mice following administration of the serotonin precursor 5-HTP [59] and was further identified to be influenced by various serotonergic-based hallucinogens. A neuropharmacological study showed that injections of 5-HT_2A_ receptor agonists into the medial prefrontal cortex of male rats caused HTR [60]. The increased levels of natural agonist of the 5-HT_2A_ receptor in the cortex might be related to the HTR in WD-fed mice. It is worth mentioning that HE may manifest extrapyramidal dysfunctions, such as involuntary body movements or dyskinesia [61,62], which can appear as head bobbing, twitching, or body swaying. Psychotic symptoms like hallucinations may appear in patients with HE [63]. The HTR is mostly regarded as a measure of drug-induced hallucinogenic effects [28,64]. However, some argue that although hallucinogens induce HTR, they do not fully represent hallucinatory behavior [65]. Whether these HTR observed in WD-fed mice correspond to the head bobbing in HE patients, or merely a behavior caused by serotonin changes, should be further evaluated, particularly if previously identified 5-HT receptors are also involved. Also, WD reduced hippocampal 5-HT, but the values did not amount to statistical significance.

Furthermore, cortical Glu and GABA were increased in WD-fed mice. The Glu and GABA represent the main excitatory and inhibitory neurotransmitters, respectively, relevant to memory, cognition, and mood regulation. Changes in Glu and GABA levels affect cortical excitability. When in excess, Glu can be toxic, causing neuronal overexcitation leading to neuronal damage/death [66]. These enhanced Glu levels may be implicated in the behavioral impairments and possibly influenced by the microgliosis in WD-fed mice. When activated by pro-inflammatory stimuli, microglia release glutamate [67], which then stimulates glutamate receptors, exerting facilitatory and inhibitory control over DA release [68] that was found reduced in WD-fed mice. Further, increased extracellular GABA in the cerebellum was linked to hypoactivity, and is related to impaired neural circuits (basal ganglia-thalamus-cortex circuits) and increased cognitive decline in liver disease [69,70]. Although we saw hypoactivity and inattention in WD-fed mice, cerebellar GABA was unchanged, suggesting other factors involved in the behavioral changes in WD-fed mice, including modulatory influences by other neurotransmitters, particularly DA suppression [71].

In conclusion, the applied protocol of 48 weeks of WD feeding represents a reproducible animal model where steatohepatitis is accompanied by specific patterns of behavioral changes, altered neurotransmitter expressions in specific brain regions, and histological changes, offering good conditions to study causal relationships and to identify therapeutic interventions in the future.

## Data Availability

The data presented in this study are available on request from the corresponding author.

## Supplementary Materials

The following supporting information can be downloaded at: https://www.mdpi.com/article/10.3390/cells12182331/s1, Table S1: Materials and sources. Table S2: Ingredients of the Western diet (WD). Table S3: Used antibodies and their concentrations.
