# Supplementary material for: Cognitive Functions, Neurotransmitter Alterations, and Hippocampal Microstructural Changes in Mice Caused by Feeding on Western Diet
Source: Cells. 2023 Sep 21;12(18):2331. doi: 10.3390/cells12182331 (PMC10529844; doi:10.3390/cells12182331)
Supplement: Supplementary file 1 [file cells-12-02331-s001.zip › cells-2609862-supplementary.pdf]

Supplementary Table S1. Materials and sources.

Materials	Source	Reference
Anti-CD31 antibody, rat	BD Biosciences, Heidelberg, Germany	550274
Anti-GFAP antibody, chicken	Covance, Princeton, NJ, USA	PCK-591P
Anti-Iba1 antibody, goat	Abcam, Cambridge, UK	ab5076
Anti-liver arginase1 antibody, rabbit	Abcam, Cambridge, UK	ab203490
Anti-mouse CD45 antibody, rat	BD Biosciences, Heidelberg, Germany	550539
Anti-CPS1 monoclonal antibody, rabbit	Abcam, Cambridge, UK	ab129076
Anti-GS polyclonal antibody, rabbit	Sigma, St. Louis, MO, USA	G2781
Bluing Reagent	Roche, Mannheim, Germany	05266769001
Chromo Map DAB	Roche, Mannheim, Germany	05266645001
Discovery OmniMap anti-rat HRP	Roche, Mannheim, Germany	05891892001
Discovery UltraMap anti-goat HRP	Roche, Mannheim, Germany	06607241001
Discovery UltraMap anti-rabbit HRP	Roche, Mannheim, Germany	05269717001
Eosin Y disodium salt	Sigma-Aldrich, St. Louis, MO, USA	E4382
Hematoxylin II	Roche, Mannheim, Germany	05277965001
Hoechst 33258	Thermo Fisher Scientific, Waltham, MA, USA	H1398
Mouse IL-6 Quantikine ELISA Kit	R&D Systems, Minneapolis, MN, USA	M6000B
Mouse IL-10 Quantikine ELISA Kit	R&D Systems, Minneapolis, MN, USA	M1000B
Mouse IL-12 p40 + IL-12 p70 ELISA Kit	Abcam, Cambridge, UK	ab100699
Mouse TNF-alpha Quantikine ELISA Kit	R&D Systems, Minneapolis, USA	MTA00B
Piccolo general chemistry 13	Hitado, Möhnesee, Germany	AB - 114 - 400 - 0029
Picrosirius Red Stain Kit	Polysciences Inc., Warrington, PA, USA	24901
GraphPad Prism 9.5.1 Software	GraphPad, La Jolla, CA, USA	RRID:SCR_002798
Ethovison XT v17.5 Software	Noldus, Wageningen, Netherlands	RRID:SCR_000441
ActiMot2	TSE systems, Chesterfield. MO, USA	N/A
Axio Scan.Z1	Zeiss, Jena, Germany	N/A
Arivis Vision4D 4.0 software	Zeiss, Jena, Germany	N/A
DISCOVERY ULTRA Automated Slide Preparation System	Roche, Mannheim, Germany	N/A
Piccolo Xpress® chemistry analyzer	Abaxis, Union City, CA, USA	N/A

Celldiscoverer 7	Zeiss, Jena, Germany	N/A
PocketChem BA PA-4140 ammonia meter	Arkray, Inc., Amstelveen, Netherlands	N/A
Ssniff R/M-H, 10 mm standard diet	Ssniff, Soest, Germany	V1534‐000
Western diet	Research Diets, New Brunswick, NJ, USA	D16022301

Supplementary Table S2. Ingredients of the Western diet (WD).

Ingredient	Grams	Kcal	%
Casein, 80 mesh	200	800	22.12
L-cystine	3	12	0.33
Maltodextrin 10	100	400	11.06
Fructose	200	800	22.12
Sucrose	96	384	10.61
Cellulose (BW200)	50	0	5.53
Soybean oil	25	225	2.76
Primex, non-trans fat	135	1215	14.93
Lard	20	180	2.21
Mineral Mix S10026	10	0	1.11
Dicalcium phosphate	13	0	1.44
Calcium carbonate	5.5	0	0.61
Potassium citrate	16.5	0	1.82
Vitamin Mix (V10001)	10	40	1.11
Choline bitartrate	2	0	0.22
Cholesterol	18	0	1.99
FD&C Yellow dye	0.05	0	0.006
**Total**	**904.05**	**4056**	**100**

Supplementary Table S3. Used antibodies and their concentrations.

Primary antibody		Secondary antibody	
**Antibody**	**Dilution**	**Antibody**	**Dilution**
Anti-liver arginase1 antibody, rabbit	1:2000	Ultra-Map anti-rabbit HRP	-

Anti-mouse CD45 antibody, rat	1:400	Omni-Map anti-rat HRP	-
Anti-CPS1 monoclonal antibody, rabbit	1:200	Ultra-Map anti-rabbit HRP	-
Anti-GS polyclonal antibody, rabbit	1:15000	Ultra-Map anti-rabbit HRP	-
Anti-serotonin antibody, goat	1:1000	Ultra-Map anti-goat HRP	-
Anti-Iba1 antibody, goat	1:100	Cy5 donkey anti-goat IgG (H+L)	1:200
Anti-GFAP antibody, chicken	1:2000	Cy2 donkey anti-Chicken	1:200
Anti-CD31 antibody, rat	1:50	Cy2 donkey anti-rat IgG (H+L)	1:200
Hoechst 33258	1:5000		
